# The sealing ability of novel Kryptonite adhesive bone cement as a retrograde filling material

**DOI:** 10.15171/joddd.2016.030

**Published:** 2016-08-17

**Authors:** İsmail Uzun, Cangül Keskin, Buğra Güler

**Affiliations:** ^1^Assistant Professor, Department of Endodontics, Faculty of Dentistry, OndokuzMayýs University, Samsun, Turkey; ^2^PhD. Dentist.Department of Endodontics,Faculty of Dentistry,Ondokuz Mayýs University, Samsun, Turkey; ^3^PhD. DentistSamsun Oral Health Clinic, Samsun, Turkey

**Keywords:** Apical microleakage, dye penetration, Kryptonite bone cement, MTA

## Abstract

***Background.*** This study evaluated the ability of Kryptonite bone cement in sealing retrograde cavities.

***Methods.*** The root canals of one hundred extracted human maxillary incisor teeth were instrumented up to master apical file #40 using Mtwo rotary system and obturated with gutta-percha and AHPlus sealer by cold lateral compaction method. The specimens were assigned to one control group and four experimental groups based on the retrograde filling materials (n=20). The specimens were immersed in 0.5% Rhodamine B solution for 48h. Then the specimens were divided longitudinally into two parts and the depth of dye penetration was assessed under ×10 magnification. Data were analyzed using one-way ANOVA and Bonferroni tests.

***Results.*** There were statistically significant difference between the experimental groups and the control group (P<0.05). There were no statistically significant differences between the experimental groups in dye penetration scores (P>0.05).

***Conclusion***. Kryptonite cement provided optimal apical seal in a manner similar to MTA, amalgam and IRM when used as a retrograde filling cement.

## Introduction

Apicoectomy and retrograde filling is applied to treat persistent endodontic infections and teeth with failed endodontic treatment.^1^ Efficacy of periapical surgery depends on infected debris and microorganism elimination and also providing an adequate apical seal.^2^ The ideal retrograde filling substance should be non-resorbable and easily manipulated, exhibitdimensional stability, biocompatibility, radiopacity and resistance to moisture. In addition, it should adapt to prepared canal anatomy, seal the root canal and encourage healing.^3^ Numerous materials, including zinc oxide-eugenol, gold foil, composite resin, reinforced zinc oxide-eugenol, mineral trioxide aggregate and calcium silicate-based cements, have been suggested and tested as root-end filling materials.^4,5^ In line with these advances, many new materials have been developed and tested.

Kryptonite bone cement (Doctors Research Group Inc, Southbury, CT, USA) is a castor oil-derived polymer adhesive, which is biocompatible and defined as “a polymer that comprises castor oil-based polyols, a reactive isocyanate and calcium carbonate powder that cures in situ”.^6^ Kryptonite has been reported to adhere directly and specifically to bone tissue, provide rigid fixation in 24 hours and prevent bone displacement in previous studies.^7,8^The adhesive has 3 components (A, B and C bottles) and 4 minutes of application time. The material reaches maximal polymerization at 24 hours.^7^The polymer ingredient has been reported as biocompatible and since its introduction in 2006, no adverse effects related to biocompatibility have been reported.^6,9^Our literature research revealed no study on the properties of Kryptonite bone cement when used on dental tissues, yet.

The aim of the present study was to evaluate the efficacy of Kryptonite in apical sealing in comparison with MTA, IRM and amalgam using dye penetration technique.

## Methods

The study protocol was approved by OndokuzMayis University Clinical Researches Ethics Board.

One hundred extracted human maxillary incisor teeth were collected, with single root canal, for this study. Teeth showing previous root canal treatment, open apices, cracks, fractures, calcifications and resorption were excluded. All the roots were cut to a length of 13 mm with a diamond bur (K6974, GEBR Brasseler GmbH and Co.KG, Lemgo, Germany) with water spray. Working length was determined visually by introducing a #10 K-file (Dentsply, Maillefer, Ballaigues, Switzerland) into the root canal until it was visible at the apical foramen and subtracting 1 mm from that measurement. The root canals were prepared with the Mtwo rotary file system (Mtwo, VDW, Munich Germany) up to #40.04 file. TwomL of 2.5% NaOClwere used for irrigation between each preparation step with 30-G NaviTip irrigation needles (Ultradent, South Jordan, UT) inserted 1 mm shorter than the working length. Final irrigation was performed with 5 mL of 17% EDTA for 1 minute and 5 mL of distilled water. Then the root canals were dried with sterile paper points (Dentsply, Maillefer) and filled using AH Plus root canal sealer (Dentsply, Konstanz, Germany)and gutta-percha by cold lateral compaction technique. Access openings were sealed with temporary filling material (Cavit G, 3M ESPE Dental AG, Seefeld, Germany). In order to achieve a complete setting of the sealing material the roots were kept at 37°C in 100% humidity for a week .

A 3-mm length of the apical end of each root was cut off perpendicular to the long axis of the root with a No.701 fissure bur. Retro-cavities were prepared to a depth of 3 mm coaxially using surgical retro-preparation tips (Satelec AS6D, France). The cavities were irrigated with sterile saline and dried with paper points. The specimens were assigned randomly to 4 experimental groups and two negative and positive control groups in terms of the retro filling material:

**Group1:** White MTA (Dentsply, Tulsa Dental Specialties, Tulsa, OK, USA).

**Group 2:** Amalgam (Cavex, Avalloy, Haarlem, Holland) was triturated according to the manufacturer’s instructions using an amalgamator (SDI Ultramat 2, Victoria, Australia).

**Group 3:** IRM (Dentsply Caulk, Milford, DE, USA).

**Group4:** Kryptonite (DoctorsResearchGroupInc, Southbury, CT, USA) was mixed as previously described by Fedak and Kasatkin.^8^ Components A, B and C were mixed for 1 minute to form a viscous liquid and left static on a glass slab for another3 minutes to gain a moldable putty form before application. The adhesive was applied into the retrograde cavity using a plastic spatula and packed into the cavity with hand pluggers.

In the positive control group,10 specimens with retro-cavities were prepared but they did not receive any retrograde filling material. Another ten specimens in the negative control group did not receive any retro-cavity preparations and retrograde filling; their root surfaces were coated with two layers of nail polish.

All the materials were placed incrementally according to the manufacturers’ instructions and control radiographs were taken to check the quality of retrograde fillings. The root surface was coated with two layers of nail polish and then allowed to be dried. The specimens were kept in 100% humidity at 37°C for 24 hours to allow complete setting or polymerization of materials.^6^The specimens were immersed in 0.5% Rhodamine B solution for 48 hours. Then the specimens were rinsed for 15min under distilled water. The specimens were grooved longitudinally in order to separate them into two halves using a separating disc. Dye penetration microleakage was evaluated under stereomicroscope at ×10 (Zeiss SV, Oberkochem, Germany) in millimeters. Data were analyzed using one-way ANOVAin SPSS software (PASW Statistics 20; SPSS Inc, Chicago, IL, USA). Further interactions and significances were investigated with post-hoc Bonferroni test. The level of statistical significance was set at P <0.05.

## Results

**Figure 1.**
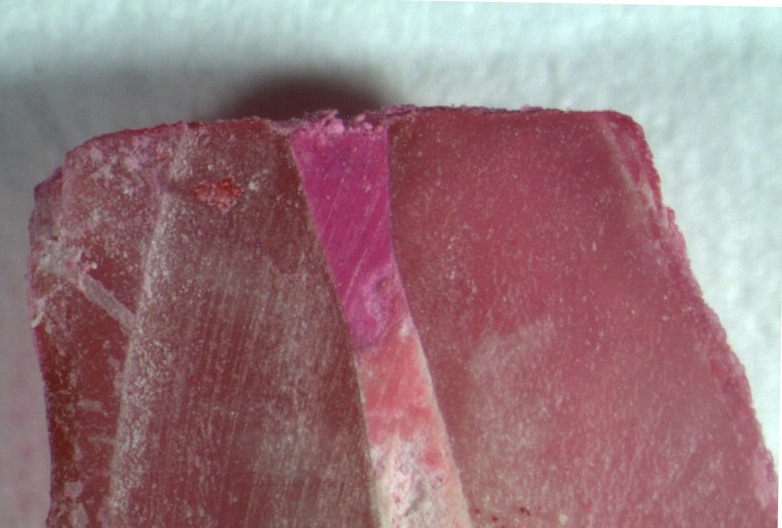
Dye penetration beyond the IRM retrograde material.

**Figure 2.**
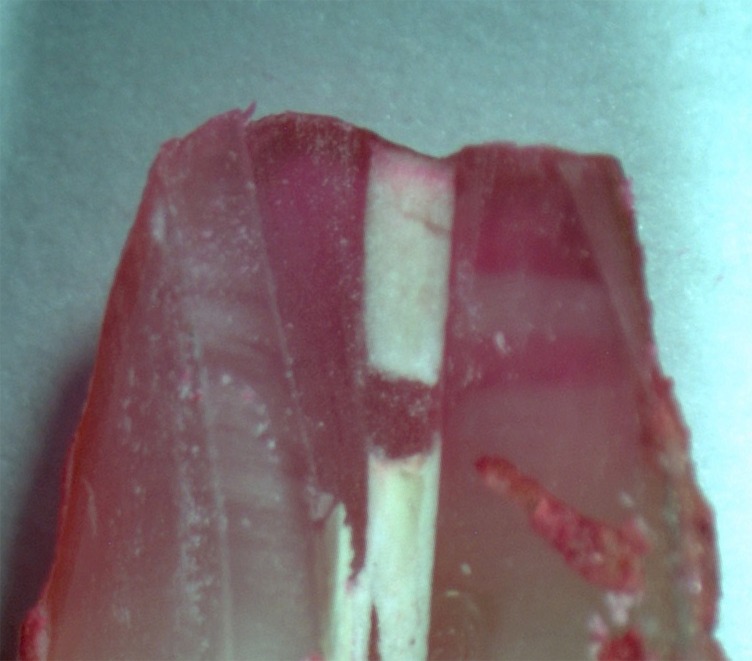
Minimal dye penetration was shown with MTA retrograde material.

**Figure 3.**
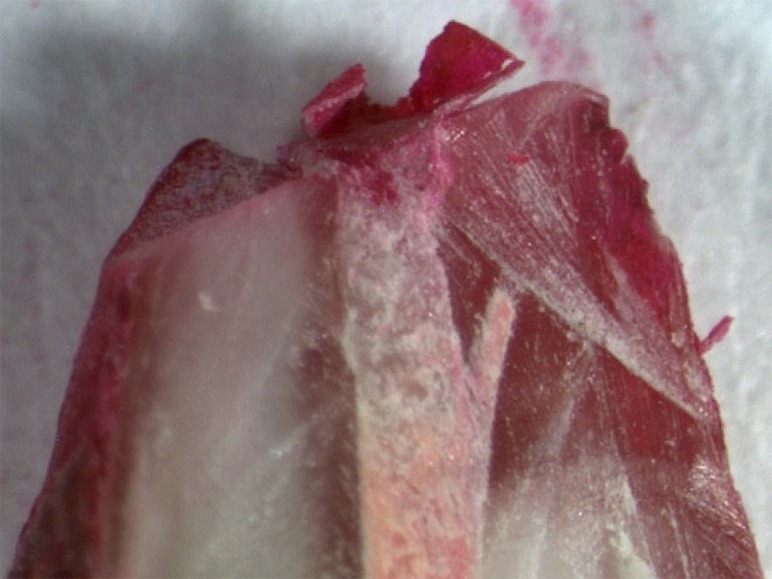
Minimal dye penetration into the Kryptonite bone cement when used as a retrograde filling material.

**Figure 4.**
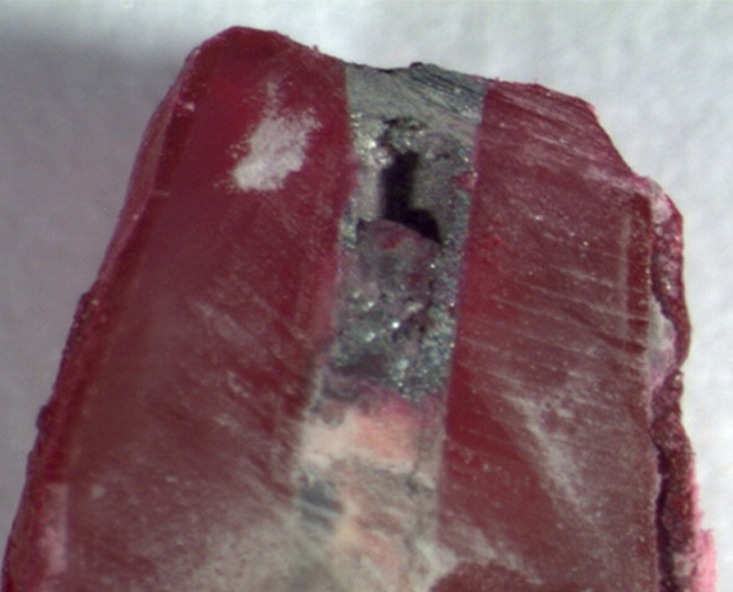
Dye penetration beyond amalgam.

All the specimens of the positive control group showed dye leakage throughout the canal lengths, whereas specimens of the negative control group revealed no dye penetration (Figures 1-4). Groups 1, 2, 3 and 4 showed statistically significant differences from the positive and negative control groups (P<0.05).

Dye penetration was detected in all the experimental groups. The mean and standard deviation values of dye penetration depths are detailed in Table 1. One-way ANOVA revealed no statistically significant differences between groups 1, 2, 3 and 4 (P>0.05).

**Table 1:** Mean and standard deviation values of dye penetration depths according to the retrograde filling materials in millimeter

	**Group 1 (MTA)**	**Group 2 (Amalgam)**	**Group 3 (IRM)**	**Group 4 (Kryptonite)**
**Dye penetration scores (mm)**	2.75 ± 0.58	3.01 ± 0.98	3.19 ± 0.74	2.98 ± 0.43

## Discussion

The effectiveness and success of endodontic surgery relies on proper root-end resection, preparation and providing an adequate seal.^2^ Retrograde filling placement aims to provide an apical seal to prevent leakage of irritant substances from the root canal to adjacent circum-radicular structures.^1^Removing of the apical 3-mm of root end has been shown to eliminate lateral canals and apical ramifications, which might contribute to endodontic failure.^2^ In the present study, the apical 3-mm of root ends were resected perpendicular to the long axis of the root. Ultrasonic preparation of retrograde cavities has been shown to be safer, as it reduces perforation risk and provides centered and controlled procedures.^10^Therefore, in the present study we used ultrasonic tips to prepare retrograde cavities.

Amalgam has been traditionally used as a retrograde filling material, but it has some disadvantages, including scattering of amalgam particles into periradicular tissues, material corrosion and dimensional instability.^1^The present study determined the sealing ability of a material by its ability to inhibit dye penetration. The results of the present study confirmed the findings of previous studies that reported that amalgam retrograde fillings showed leakage.^11,12^ Schwartz and Alexander suggested that cavity varnish application on amalgam surface improves the sealing ability of the material.^13^

IRM is a modified zinc oxide eugenol and has been considered an alternative retrograde filling material to amalgam. IRM has been reported to show less leakage as compared to amalgam.^14^ Another study investigated the clinical performance of IRM in retrograde filling and reported 91.2% success rate both clinically and radiographically.^15^ IRM is also reported to demonstrate comparable clinical success rates with MTA.^11^ The present study reported that the sealing ability of IRM was lower than that of MTA, but this difference was not statistically significant.

MTA forms a colloidal gel when freshly mixed with water and this gel solidifies within 4 hours.^16^When MTA interacts with tissue fluids, the physiochemical reaction results in the formation of hydroxyapatite, which is responsible for MTA’s sealing ability.^17^Despite the long setting time, MTA has became the most popular retrograde filling material as a result of higher sealing ability compared to amalgam and zinc oxide eugenol-based cements.^18,19^In the present study, MTA exhibited the highest sealing ability, but no statistically significant difference was reported.

The present study found that Kryptonite provides apical sealing as well as MTA. Dye leakage scores of Kryptonite were lower than amalgam and IRM; however, the difference was not statistically significant. Kryptonite was developed as radiopaque bone adhesive cement and its use on dental tissues has not been reported yet. Kryptonite was initially used for rigid sternal fixation and was reported to show resistance to displacement forces less than 600 Newtons.^7^ Kryptonite also possesses osteoconductive properties and does not interfere with normal healing.^8^ Animal and in vitro cell studies have shown osseointegration within the adhesive cement.^20^The exact mechanism for its superior sealing ability has not been studied yet. Kryptonite constitutes 3 components: the first component is a castor oil-derived polymer; the second component is a polyol, which contains hydroxyl-terminated fatty acid, water and catalyst. The third component, calcium carbonate, is held responsible for osteoconductive properties by providing mechanical strength, structure and acting as a scaffold.^21^Among the tested materials, MTA and Kryptonite have osteoconductive properties and react with tissues. As a retrograde filling material Kryptonite might have some certain advantages over amalgam such as sealing ability and osteoconductive properties.

Dye penetration studies assume that a material, which does not get penetrated by small molecules, prevents leakage of larger substances such as microorganisms and their byproducts.^1^Therefore, the results of dye penetration studies are difficult to directly relate with clinical applications. But dye penetration study protocol provides a cheap, easy to conduct and quantitative method to measure leakage.^22^ Rhodamine B was selected for its stable and durable features in different conditions, as emphasized and suggested by Rahimi et al.^23^ Within the limitations of the present study, Kryptonite provided apical seal comparable to that of MTA, amalgam and IRM when used as a retrograde filling material. Further studies are required to investigate mechanical, chemical and biological properties of this novel material and its suitability for in vivo use.

## Authors’ contributions

İU was responsible for the concept and design of the work, and drafting the manuscript.CK was responsible for acquisition, analysis, or interpretation of data, drafting and writing the manuscript.BG was responsible for the design of the work, preparation of samples and experiments and revising the manuscript critically. All the authors have read and approved the final manuscript.

## Funding

No funding is reported.

## Competing interests

The authors declare no competing interests with regards to the authorship and/or publication of this article.

## Ethics approval

The OndokuzMayis University Clinical Researches Ethics Board approved the study protocol.

## References

[1] Aqrabawi J (2000). Endodontics: Sealing ability of amalgam, super EBA cement, and MTA when used as retrograde filling materials. British Dent J.

[2] Kim S, Kratchman S (2006). Modern endodontic surgery concepts and practice: a review. J Endod.

[3] Gartner A, Dorn S (1992). Advances in endodontic surgery. Dent Clin North Am.

[4] Dorn SO, Gartner AH (1990). Retrograde filling materials: a retrospective success-failure study of amalgam, EBA, and IRM. J Endod.

[5] Torabinejad M, Watson T, Ford TP (1993). Sealing ability of a mineral trioxide aggregate when used as a root end filling material. J Endod.

[6] Doctors Research Group (2009). Kryptonite bone void filler- biocompatibility and osseointegration.

[7] Fedak PW, Kolb E, Borsato G, Frohlich DE, Kasatkin A, Narine K (2010). Kryptonite bone cement prevents pathologic sternal displacement. Annals Thorac Surg.

[8] Fedak PW, Kasatkin A (2011). Enhancing Sternal Closure Using Kryptonite Bone Adhesive Technical Report. Surg Innov.

[9] DesLauriers, RJ, Kolb E, Boxberger J. Polymeric bone defect filler. U.S. Patent No. 8,338,498. 25 Dec. 2012.

[10] Engel TK, Steiman HR (1995). Preliminary investigation of ultrasonic root end preparation. J Endod.

[11] Gerhards F, Wagner W (1996). Sealing ability of five different retrograde filling materials. J Endod.

[12] Chong B, Ford TP, Watson T (1991). The adaptation and sealing ability of light‐cured glass ionomer retrograde root fillings. Int Endod J.

[13] Schwartz SA, Alexander JB (1988). A comparison of leakage between silver-glass ionomer cement and amalgam retrofillings. J Endod.

[14] Smee G, Bolanos OR, Morse DR, Furst ML, Yesilsoy C (1987). A comparative leakage study of P-30 resin bonded ceramic, Teflon, amalgam, and IRM as retrofilling seals. J Endod.

[15] Lindeboom JA, FrenkenJW FrenkenJW, Kroon FH, van den Akker HP (2005). A comparative prospective randomized clinical study of MTA and IRM as root-end filling materials in single-rooted teeth in endodontic surgery. Oral Surg Oral Med Oral Pathol Oral Radiol Endod.

[16] Torabinejad M, White DJ. Tooth filling material and use. United States patent number 5,769,638:1995.

[17] Sarkar NK, Caicedo R, Ritwik P, Moiseyeya R, Kawashima I (2005). Physicochemical basis of the biologic properties of mineral trioxide aggregate. J Endod.

[18] Torabinejad M, Rastegar AF, Kettering JD, Ford TRP (1995). Bacterial leakage of mineral trioxide aggregate as a root-end filling material. J Endod.

[19] Mangin C, Yesilsoy C, Nissan R, Stevens R (2003). The comparative sealing ability of hydroxyapatite cement, mineral trioxide aggregate, and super ethoxybenzoic acid as root-end filling materials. J Endod.

[20] Adams DJ, Barrero M, Jiang X, Rowe DW (2008). Persistent osteoconductivity of calcium triglyceride bone cement in osteoporotic bone. Transac 54th Annual Meet Orthopaedic Res Soc.

[21] Bre LP, Zheng Y, Pego AP, Wang W (2013). Taking tissue adhesives to the future: from traditional synthetic to new biomimetic approaches. Biomater Sci.

[22] Wu MK, Wesselink PR (1993). Endodontic leakage studies reconsideredPart IMethodology, application and relevance. IntEndod J.

[23] Rahimi S, Asgary S, Samiei M, Bahari M, Pakdel SMV, Mahmoudi R. The effect of thickness on the sealing ability of CEM cement as a root-end filling material. J Dent Res Dent Clin Dent Prospects 2015;9,1-6. 10.15171/joddd.2015.002 PMC4417497 25973147

